# New value chain *Pentadesma* nuts and butter from West Africa to international markets: Biological activities, health benefits, and physicochemical properties

**DOI:** 10.1002/fsn3.3806

**Published:** 2023-11-20

**Authors:** Ifagbémi Bienvenue Chabi, Midimahu Vahid Aïssi, Oscar Zannou, Yénoukounmè E. Kpoclou, Bernolde Paul Ayegnon, Marius Eric Badoussi, Vénérande Y. Ballogou, Gulden Goksen, Amin Mousavi Khaneghah, Adéchola P. Polycarpe Kayodé

**Affiliations:** ^1^ Laboratory of Human Nutrition and Valorization of Food Bio‐Ingredients, Faculty of Agricultural Sciences University of Abomey‐Calavi Cotonou Benin; ^2^ Laboratoire de Science et Technologie des Aliments et Bioressources et de Nutrition Humaine, Ecole des Sciences et Techniques de Conservation et de Transformation des Produits Agricoles Université Nationale d'Agriculture Sakété Benin; ^3^ Ecole Nationale Supérieure des Biosciences et Biotechnologies Appliquées Université Nationale des Sciences Technologies Ingénierie et Mathématiques Abomey Benin; ^4^ Unité de Recherche en Génie Enzymatique et Alimentaire, Laboratoire d'Etude et de Recherche en Chimie Appliquée, Ecole Polytechnique d'Abomey‐Calavi Université d'Abomey‐Calavi Cotonou Benin; ^5^ Department of Food Technology, Vocational School of Technical Sciences at Mersin Tarsus Organized Industrial Zone Tarsus University Mersin Turkey; ^6^ Department of Fruit and Vegetable Product Technology Prof. Wacław Dąbrowski Institute of Agricultural and Food Biotechnology, State Research Institute Warsaw Poland

**Keywords:** biological properties, butter tree, nutritional composition, phytochemicals, vegan butter

## Abstract

The tallow or butter tree (*Pentadesma butyracea* Sabine) is a ligneous forest species of multipurpose use largely distributed in Sub‐Sahara Africa. Owing to the biological properties of different parts of the tree and physicochemical properties, as well as the numerous benefits of its fruits, research on *P. butyracea* products, especially kernels and butter, has now gained more interest. Thus, the scientific literature revealed that *Pentadesma* butter is a more promising product with good physical and technological characteristics. It is traditionally preferred in households for food, medicine, and cosmetic use. Apart from the fruits, all other parts of the butter tree are used by local communities in folk medicine. The existing studies indicated that *P. butyracea* contains valuable health‐promoting compounds such as phenolic compounds, vitamins, minerals, and essential fatty acids. *P. butyracea* and derived products have antioxidant, antimicrobial, anti‐inflammatory, antiplasmodial, antitumor, estrogenic, anti‐androgenic, and cholesterol‐regulative effects. Since studies on the biological properties of the tree parts, nutritional composition, and physicochemical properties of food products from the tree have been very limited, this review attempts to summarize some results from recent investigations. Our intention in the present review was to give an overview of the biological activities of plants and an account of the potential properties of *Pentadesma* products (pulp, kernels, and butter) and outline the way for future relevant research to improve their state of knowledge.

## Graphical abstract

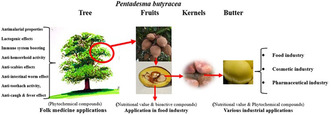

## 1 INTRODUCTION

Forest products contribute to the food security and health promotion of millions of people around the world while providing substantial resources to the national economy (Aissi et al., 2011; Amiri et al., 2021; Avocèvou‐Ayisso et al., 2009; Ayegnon et al., 2021a). Lately, using quantitative ethnobotanical methods, local communities' knowledge has been investigated to identify promising plants regarding nutritional quality, medicinal properties, and/or commercial potential (Dembélé et al., 2015). Using these promising plants could contribute to food security by improving the livelihoods of local populations (Amiri et al., 2021; Dembélé et al., 2015). Local communities in West Africa and Biodiversity International selected ten forest tree species with food value as priority species (Vinceti et al., 2022). Among these, the shea tree (*Vitellaria paradoxa*) and the butter tree (*Pentadesma butyracea*) are two of the top ten agroforestry tree species that supply butter. However, industrial interest in fats as raw materials resides in their exceptional quality and potential for exploitation in several fields (Megnanou & Niamke, 2015; Timtey et al., 2023). Shea butter is one of the most beneficial fats produced by plants. Due to its physicochemical properties, this valuable product has gained importance in the multi‐billion‐dollar international confectionery and cosmetics markets (Glew & Lovett, 2014). Shea has been described as an important tree in socioeconomic terms and was included in the FAO's priority list of African genetic resources (Allen et al., 2019). Due to the requirements of industrialists who consider many properties, using shea butter as a raw material (for the confectionery, cosmetic, and pharmaceutical industries) could be linked to its exceptional quality and high exploitation potential (Honfo et al., 2014). Currently, the exportation of shea is estimated to be 350,000 tons of shea equivalent (Iddrisu et al., 2019). This is multiplied by recent prices per gross tonnage, which can cost rural communities in the West African region $150 million annually. This activity contributes up to 12% of the total income of the poorest households (Iddrisu et al., 2019). Chocolate and confectionery products account for about 90% of shea butter demand, while only 10% are used for cosmetics and pharmaceuticals (Bello‐Bravo et al., 2015). As can be noticed, this product is very attractive in the local, regional, and international marketplaces. Like shea kernels, *Pentadesma* kernels are the basis for *Pentadesma* butter, and both shea and *Pentadesma* kernels might find their way onto local, regional, and international markets. Unfortunately, *Pentadesma* kernels and their butter, unlike shea products (kernels and butter), were not known for the same success despite their potential application in the food, pharmaceutical, and cosmetic industries (Timtey et al., 2023). Therefore, what are these potentials? Over the past two decades, several research efforts have been carried out to increase knowledge about the nutritional quality and physicochemical properties of *Pentadesma* fruits and their products (mesocarp, kernels, and butter). *Pentadesma* is a rich source of nutrients and bioactive compounds such as phenolic and essential fatty acids. It was revealed that bioactive compounds are health‐promoting compounds (El‐Nashar et al., 2022). For the most part, the results of these works were scattered. They did not enable us to participate effectively in promoting *Pentadesma* products, especially *Pentadesma* butter, whose quality would be better than shea butter. Additionally, the detailed and comprehensive reviews of the scientific data available on shea trees, kernels, and butter (Honfo et al., 2014; Tom‐Dery et al., 2018) greatly increase the visibility and economic value of shea products. Despite its nutritional, cosmetic, pharmaceutical, and socioeconomic importance, to the best of our knowledge, no detailed review has been devoted to *Pentadesma* products until now. However, the aim of this paper is not to present a comprehensive review of *P. butyracea* products but to gather essential scattered information on the tree and its ethnobotanical importance, with a particular emphasis on the nutritional value and physicochemical properties of *Pentadesma* kernels and butter, and to highlight the health benefits and prospects of *P. butyracea* products.

## 2 METHODOLOGY OF DATA COLLECTION

A comprehensive literature search was performed by combining the appropriate keywords, including “tallow tree,” “butter tree,” “*Pentadesma butyracea* Sabine,” “taxonomy,” “nutritional composition,” “proximate composition,” “traditional uses,” “phytochemicals,” “physiochemical properties,” “antioxidant properties,” “bioactive compounds,” “health benefits.” As for the search engine, Google Scholar, Scopus, Web of Science, ScienceDirect, and PubMed were independently searched (Aranega & Oliveira, 2022; De Souza et al., 2021; Huang et al., 2022; Tian et al., 2023). Both English and French‐language published articles were considered. There was no year restriction, and the final search was conducted on February o5, 2023. The references were managed with Mendeley software.

## 3 TAXONOMIC CLASSIFICATION AND GEOGRAPHICAL DISTRIBUTION OF *PENTADESMA BUTYRACEA*

As shown in Table 1, the genus *Pentadesma* is the flowering plant belonging to Clusiaceae (Guttiferae), one of the 42 families of the order Malpighiales. To date, only four African species of this genus, including *P. butyracea* Sabine, *P. grandifolia* Baker, *P. lebrunii* Staner, and *P. renders* Spirlet, are reported in the literature (Ewédjè et al., 2012). However, Djoufack et al. (2010) state that *P. reyndersii* and *P. granadifolia* refer to the same species. This suggests the real existence of three different species. In a more recent publication, Tala et al. (2013) enumerated fifteen species from the genus Pentadesma without naming them. The multiplicity of synonyms of this taxon would result from the description of this plant in restricted collections during the colonial period by various authors (English, French, and German) who have communicated very little between them (Lankoandé et al., 2019; Sinsin & Avocèvou, 2007). According to Ewédjè et al. (2012), all species are known for their seeds yielding significant edible fat. However, *P. butyracea* is the only species described in the literature for the quality of its butter.

**TABLE 1:** Taxonomical classification of butter tree (*Pentadesma butyracea*).

Taxonomy	Names
Suprerkingdom	Corticata
Kingdom	Plantae
Subkingdom	Viridiplantae
Phylum	Tracheophyta
Class	Spermatopsida
Superorder	Rosanae
Order	Malpighiales
Family	Clusiaceae
Subfamily	Clusioideae
Genus	*Pentadesma*
Species	*butyracea*

**FIGURE 1:**
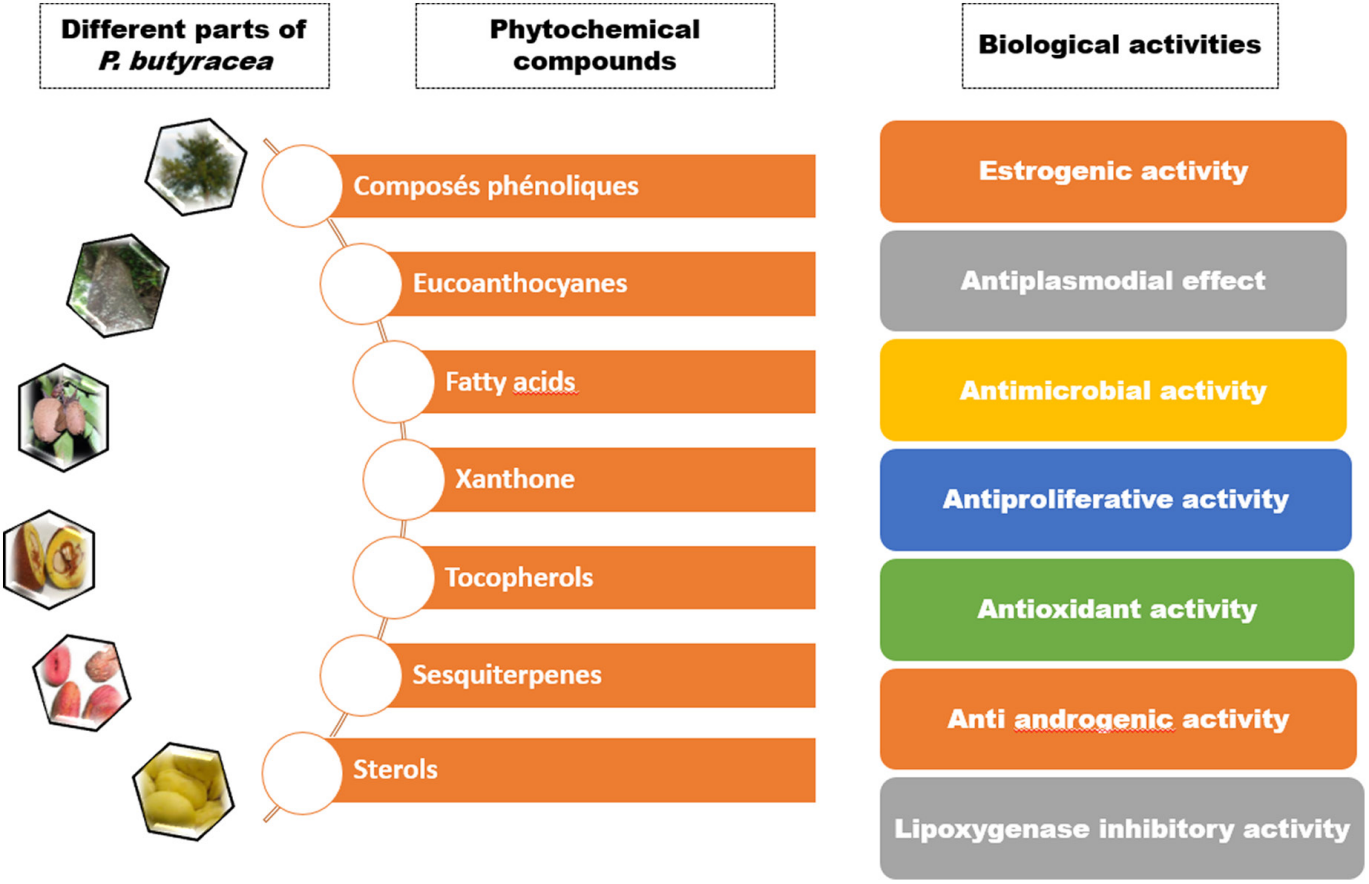
Phytochemical compounds and biological activities of *P. butyracea*.

Moreover, the geographical distribution reported in the literature was based on the four species mentioned earlier. According to that, *P. renders* Spirlet species endemic to Rwanda, while *P. grandifolia* Baker is only found in the region, including Nigeria, Cameroon, and Gabon. *P. lebrunii* Staner species is native to the Democratic Republic of Congo and Burundi. The last one, *P. butyracea* Sabine (Figure 1), has the widest distribution area and is the most valuable in socioeconomic terms. Other species should be more scientifically investigated. The geographical distribution of this species extends from Guinea‐Bissau to the Democratic Republic of the Congo (Ewédjè et al., 2012), with an extension toward the East. However, Sama et al. (2007) reported that the presence of *P. butyracea* Sabine in East Africa, especially in Tanzania and Uganda, was under cultivation. *P. butyracea* Sabine, commonly known as the butter tree or tallow tree, is an evergreen tree that grows mainly in forests and can reach 35 m in height.

## 4 MEDICINAL POTENTIAL AND BIOLOGICAL PROPERTIES OF PHYTOCHEMICALS OF *P. BUTYRACEA* TREE

### 4.1 Medicinal uses of *P. butyracea* Sabine

Several ethnobotanical studies have shown that the different organs of the tree, especially flowers, seeds, leaves, bark, stem, and roots, are used by local communities to prevent or treat various diseases, including fever, coughs, malaria, toothaches, genitourinary infections, constipation, digestive disorders, scabies, and others. According to Sinsin and Sinadouwirou (2003) and Natta et al. (2010), the leaves are used as an antimalarial remedy and a dressing after circumcision. They are also used as a lactogenic vegetable for nursing mothers. Their consumption by the nursing mother is supposed to strengthen the immune system of their newborns as well. The fruits treat digestive disorders, genitourinary infections, and human food (Avocèvou et al., 2008). The infusion obtained from the roots is used to fight against intestinal worms, and that of the bark of the trunk is used to treat diarrhea and hemorrhoids (Akoegninou et al., 2006). In addition, it has been reported that the macerated bark and the decoction of the roots are used for treating parasitic skin diseases and as an antidiarrheal. The seeds are used to fight against scabies, breast pain, and digestive and respiratory disorders (Ambé, 2001; Avocèvou et al., 2008; Natta et al., 2010; Sinsin & Sinadouwirou, 2003).

### 4.2 Biological properties of phytochemicals from *P. butyracea* Sabine

The biological properties of butter trees are due to the numerous bioactive compounds in different tree parts (Figure 1). *P. butyracea* contains the major phytochemical classes, including terpenoids, alkaloids, and phenolics (Noudogbessi et al., 2013; Tindano et al., 2017). They are secondary metabolites biosynthesized in all tree parts, including roots, stems, barks, leaves, flowers, fruits, and seeds. Nevertheless, like most medicinal plants, the bioactive compounds of the butter plant are unevenly distributed in different parts of the tree (Ezin & Chabi, 2023). Various bioactive compounds of the classes mentioned above displayed impressive therapeutic applications, including antimicrobial, anti‐inflammatory, antiplasmodial, antitumor, estrogenic, and anti‐androgenic (Table 2). Xanthones are phenolic compounds known for their significant antioxidant activity and health benefits. They are the most studied bioactive compounds of the butter tree. Zelefack et al. (2009) isolated and characterized four new butyraxanthones (A, B, C, and D) from the tree stem bark. All the new bioactive molecules identified demonstrated cytotoxic activity against human cancer cells (MCF‐7), while only three (except butyraxanthone D) exhibited potent antiplasmodial activities against chloroquine‐resistant *Plasmodium falciparum*. Similarly, the fruit pericarp extract showed significant antiplasmodial activity against *P. falciparum* W2, the most highly resistant strain (Tajuddeen & Van Heerden, 2019). The prenyl xanthone pentadexanthone, another xanthone newly isolated from the fruit pericarp, was responsible for this activity. Lupeol is one of the main triterpenes synthesized by the butter tree (Lenta et al., 2011). Several health effects of lupeol, including antimicrobial, anti‐inflammatory, antimalarial, anti‐proliferative, antiprotozoal, anti‐invasive, and colonic healing effects, have been reported in the literature (Kasinathan et al., 2018; Lenta et al., 2011; Siddique & Saleem, 2011). Sesquiterpenes are another terpene subclass significantly present in the plant (Noudogbessi et al., 2013). The β‐caryophyllene is a bicyclic sesquiterpene lactone found in all plant parts. It is a major active component of plant origin.

**TABLE 2:** Summary of promising biological effects derived from part of *P. butyracea.*

Plant part extract	Bioactive compounds	Potential effects	References
Seeds	Tannins, phytosterols, phenolic compounds, leucoanthocyanes, and fatty acids	Estrogenic and anti‐androgenic activities	Tindano et al. (2017)
Fruit (pericarp)	Pentadexanthone, cratoxylone, α‐mangostin, and garcinone E	Antiplasmodial effect against *Plasmodium falciparum* chloroquine‐resistant strain W2	Lenta et al. (2011) Tajuddeen and Van Heerden (2019)
Fruit	Phenolic compounds	Anticancer activity and antimicrobial activity	Tamokou et al. (2013)
Leaves	Globuxanthone and 30‐epi‐cambogin	Antiproliferative activity against human cancer cell lines (BGC‐823 and Hela)	Tala et al. (2013)
	Sesquiterpenes	Antioxidant activity	Alitonou et al. (2010)
Stem bark	Xanthones	Antiplasmodial activity against a *Plasmodium falciparum* chloroquine‐resistant strain, cytotoxicity against a human breast cancer cell line (MCF‐7)	Zelefack et al. (2009)
	Xanthones and triterpenoids	Inhibitory effect on soybean lipoxygenase activity	Alitonou et al. (2010)
	Sesquiterpenes	Antioxidant activity	Alitonou (2010)
Root	Butyraxanthone E and 30‐epi‐cambogin	Antiproliferative activities against Drosophila S2 cells and two human cancer cell lines, THP‐1 (leukemia) and HCT116 (colon cancer)	Wabo et al. (2010)
	Sesquiterpenes	Antioxidant activity	Alitonou et al. (2010)

## 5 CHEMICAL COMPOSITION OF *P. BUTYRACEA* FRUIT

### 5.1 Physicochemical and nutritional composition of *P. butyracea fruit pulp*

**FIGURE 2:**
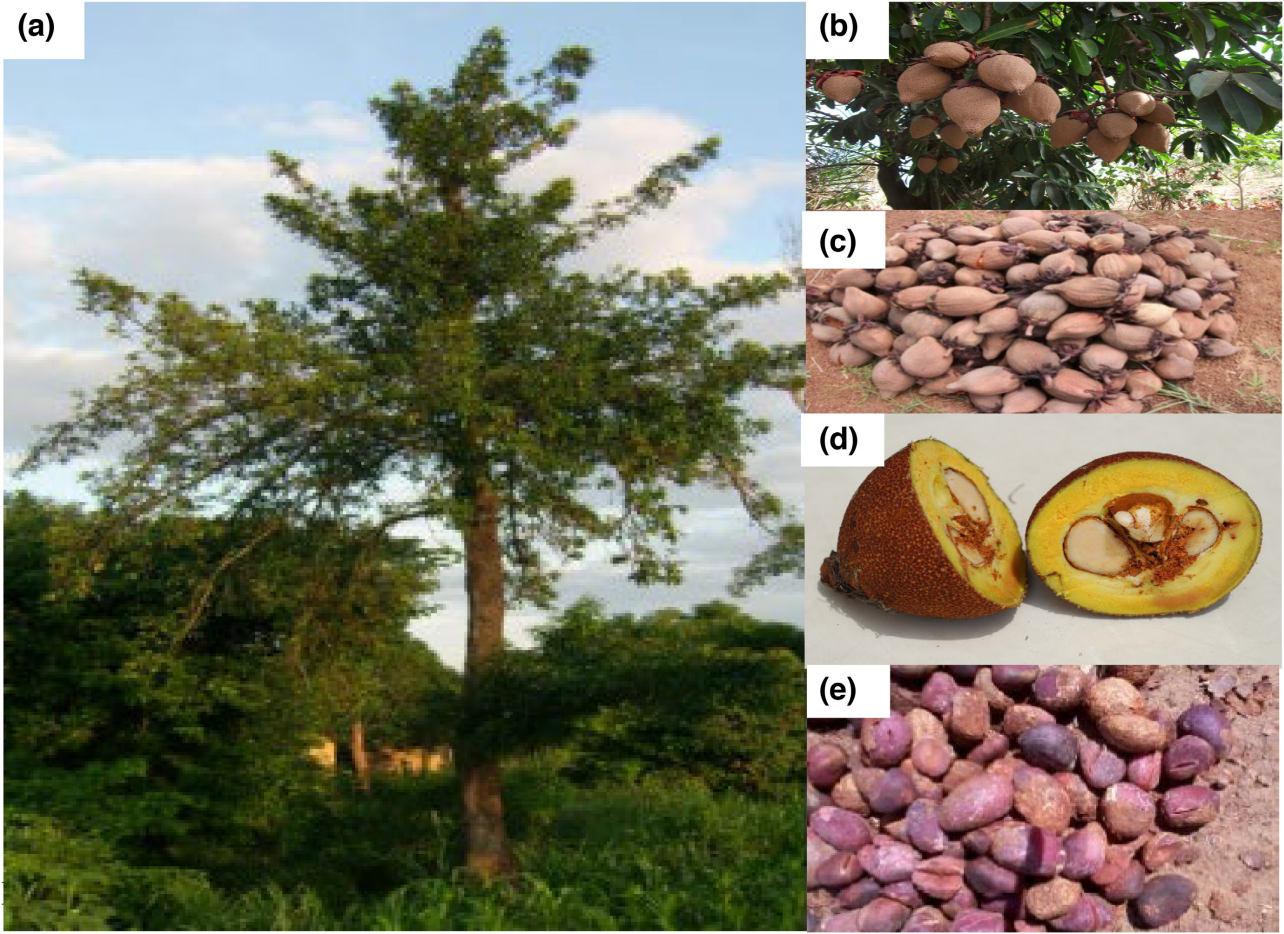
*Pentadesma butyracea* tree (a), branches with fruits (b), a heap of fruits (c), *Pentadesma* fruit pulp (d), and a heap of kernels fruit (e).

The tallow tree (Figure 2a) is mainly utilized for its kernels, which contain edible fats, and has branches with fruits (Figure 2b). Local communities also use it as a cosmetic and medicinal substance. The fruit (Figure 2c) comprises the kernels (Figure 2e), which are directly covered with yellow pulp (Figure 2d). The latter plays an important nutritional role for women, who ensure the collection of kernels. Data on the nutritional quality of this pulp are very scarce. Ayegnon et al. (2021b) reported work that is the only reference available on *Pentadesma* fruit pulp. According to this report, *Pentadesma* fruit pulp is a sour, fleshy substance (pH 3.3 and titratable acidity of 0.85%), representing about 39% of the total weight of fruit. Data on the physicochemical characteristics and bioactive properties of *P. butyracea* fruit pulp are summarized in Table 3. The water content of *Pentadesma* fruit pulp (86%) is similar to that of avocado (86.7%) but higher than that of shea (75%) fruit pulp (Honfo et al., 2014; Morais et al., 2017). The fruit pulp provides proteins (4%), lipids (6.4%), carbohydrates (66.5%), and dietary fiber (20.5%) as major macronutrients. The dietary fiber content (20.5%) of fruit pulp was twice lower than that of shea (42.2%) and avocado (41.1%). Except for the dietary fiber content, it can be assumed that *Pentadesma* fruit pulp has a higher macronutrient content than shea pulp. It is also rich in micronutrients. The fruit pulp has approximately 3% of the total ash content. However, the ash content values reported for shea pulp (5.1%) and cocoa pulp (7.6%) are much better (Honfo et al., 2014; Martínez et al., 2012). As for other fruit pulps (cocoa and shea pulps), calcium is one of the most abundant minerals in *Pentadesma* fruit pulp (Afoakwa et al., 2013; Ayegnon et al., 2021b; Honfo et al., 2014). *Pentadesma* fruit pulp is richer in iron than shea and cacao fruit pulp and contains a significant amount of zinc. Data on the vitamin content of the pulp were not available in the literature. However, this pulp's high acidity and yellow coloring (Figure 2d) could indicate a significant presence of ascorbic acid and β‐carotene. Apart from macro and micronutrients, the pulp is an important source of bioactive compounds, as shown by the value of the total phenolic content (Table 3) and that of the antioxidant activity (Ayegnon et al., 2021b).

**TABLE 3:** Nutritional composition of *Pentadesma butyracea* pulp and kernel.

	Pulp			References	Kernel			References
	Min.	Ave.	Max.		Min	Ave	Max	
Macronutrients (%)								
Moisture	85.5	–	86.4	Ayégnon et al. (2021a,b)	5	7.4	8	Ayegnon et al. (2015), Aissi et al. (2011), Kouadio et al. (1990), Adomako (1977)
Carbohydrates	65.1	–	66.9		40	43.1	45	Tchobo et al. (2013)
Crude protein	3.3	–	4.6		4	5.4	7.3	Aïssi et al. (2018), Tchobo et al. (2013), Kouadio et al. (1990)
Crude lipid	6.3	–	6.5		40	45.8	53.1	Kouadio et al. (1990), Aïssi et al. (2018)
Crude fiber	20.4	–	20.8		–	5.5	–	
Ash	2.8	–	3.6		1.8	2.8	4.3	
Energy (kcal/100 g dw)	335.8		339.0					
Micronutrients (μg/g)								
P	–		–		4.7	102.4	200	
Ca	1866.8	–	1921.8		27.7	163.9	300	Aïssi (2012), Kouadio et al. (1990), Aïssi et al. (2018)
K	–	–	–		400	950	1500	
Mg	–	–	–		99.5	300	500	
Mn	40.7		50.3		–	–	–	
Fe	176.4		205.5		0.05	0.06	0.1	
Zn	16.8		21.5		1.2	1.5	1.9	
Total phenolic (mg/ml)	21.5	‐	23.5		1.2	1.4	1.6	Tchobo et al. (2013)

### 5.2 Physicochemical and nutritional composition of *Pentadesma* kernels

The lipid and fatty acid content of *Pentadesma* kernels has been extensively studied in recent years. However, there needs to be more published information on the quantitative nutritional composition of *Pentadesma* kernels. The data available on *P. butyracea* kernels (Figure 2e) are summarized in Table 3. The moisture content of the raw kernels of *Pentadesma* is 7.4% (Aissi et al., 2011). This value follows the standard values (≤10%) recommended by the Codex Alimentarius Commission (Codex Alimentarius Commission, 1999). It was found that the fat content varies significantly between accessions and ranges from 40% to 53.1% (Table 3). The variation in levels of fat content in plant kernels could be attributed to different factors such as environmental influences, agronomic and genetic characteristics, period of harvest, and extraction methods (Grundy et al., 2020; Honfo et al., 2014; Nahm et al., 2013). Ayegnon et al. reported that the germ fraction of the *P. butyracea* kernel is the most oil‐rich part (53.15%), followed by albumen (37.49%) (Ayegnon, Kayode, et al., 2015). The level of *P. butyracea* kernel oil was higher than those of most conventional oils such as soybean (18% to 22%) (Yao et al., 2020), palm fruit (20%), palm kernel (36%), cottonseed (35%), groundnut (42%) (Jahurul et al., 2022; Ladele et al., 2016), and sunflower seed (36% to 50%) (Rauf et al., 2017), but lower than that of *Terminalia catappa* (61.76%) (Jahurul et al., 2022; Ladele et al., 2016). This level is also comparable to the shea kernel (45.2%), as reported by Honfo et al. (2014). However, it was reported that defatted kernels of *Pentadesma* contain more carbohydrates (44.84%) (Kouadio et al., 1990) than shea kernels (34.8%) (Honfo et al., 2014). Most kernel oil cakes have a relatively low protein content and are poor in essential amino acids (Prandi et al., 2019). Noudogbessi et al. (2013) investigated the nutritional profile of defatted kernels of *P. butyracea*. They found it had protein 7.3%, crude lipid 2.1%, ash 4.1%, neutral detergent fiber (NDF) 39.3%, acid detergent fiber (ADF) 29.7%, and lignin 4.9%. The ash content (2.1%) of the *Pentadesma* kernels (Adomako, 1977) was close to that reported (2.5%) by Honfo et al. (2014) in shea kernels. Regarding the microelements, Aïssi (2012) reported that zinc (Zn), iron (Fe), chloride (Cl), and sodium (Na) content in *P. butyracea* kernels were 1.86 mg/100 g, 0.055 mg/100 g, 138.09 mg/100, and 25.47 mg/100 respectively. The levels of Fe and Na content were lower than those (3.1 mg/100 g, 73.9 mg/100 g, respectively) found by Megnanou et al. (2007) in the shea (*Vitellaria paradoxa*). According to Tchobo et al. (2013), the most important minerals in *Pentadesma* kernels were potassium (1.33%), magnesium (0.35%), phosphorus (0.21%), and calcium (0.14%). Tom‐Dery et al. (2018) reported similar values for calcium in shea kernels. However, the values of potassium and magnesium reported by Noudogbessi et al. (2013) and Tchobo et al. (2013) in *Pentadesma* kernels were higher than those of Tom‐Dery et al. (2018) in shea kernels. Potassium is the more abundant element, with 50% to 75% of the total identified minerals in *Pentadesma* kernels (Tchobo et al., 2013). To our knowledge, no referenced data is available on the vitamin content of *Pentadesma* kernels. Similarly, no author has investigated the vitamin content of shea kernels. Moreover, like other vegetable products, *Pentadesma* kernels have some antinutritional substances. Although few studies readily available have investigated these substances, Tchobo et al. (2013) reported the presence of antinutritional factors such as polyphenolic compounds, phytate, and oxalate in *Pentadesma* kernels.

## 6 NUTRITIONAL COMPOSITION OF *PENTADESMA* BUTTER

**FIGURE 3:**
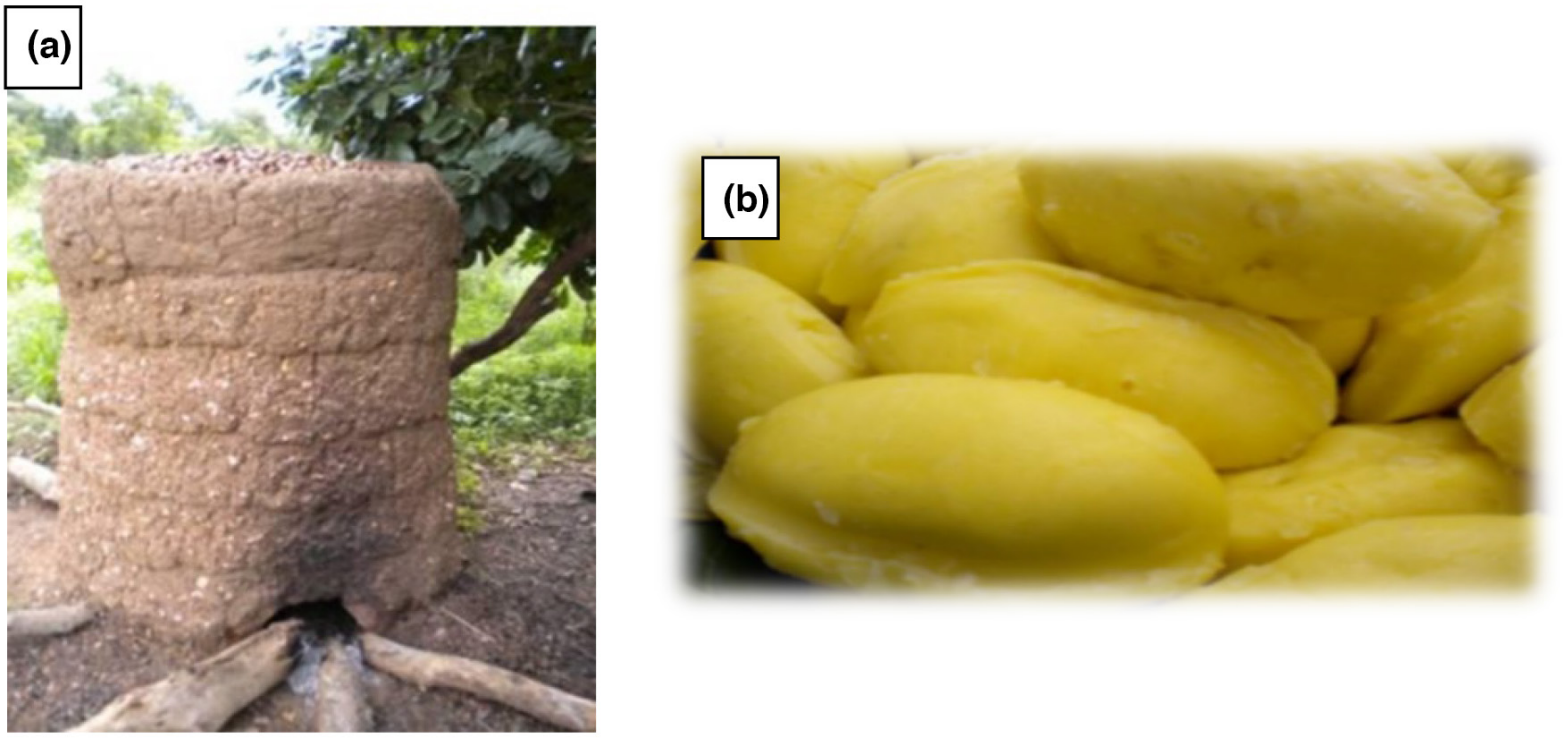
(a) Traditional kiln used for *Pentadesma* kernels roasting, and (b) some pieces of *Pentadesma* butyracea butter.

Recently, a field investigation showed that butter consumers are willing to pay more for butter with good nutritional quality (Saulais & Ruffieux, 2012). However, studies dealing with the proximate nutritional content of *Pentadesma* butter (Figure 3b) are very scarce compared to those performed on shea butter. Available information on this butter showed that water content was 0.65% (Ayegnon, Kayodé, et al., 2015) against 4.9% for shea butter (Honfo et al., 2011). Nevertheless, the low water content (1.37%) of shea butter has been reported by Chukwu Adgidzi (Chukwu & Adgidzi, 2008). Depending on their water content, the Codex Alimentarius has classified two types of unrefined butter: (i) grade I butter (for direct consumption, pharmaceutical, and cosmetic industries), which must have a water content of 0.05% or less, and (ii) grade II butter (for the food industry), which must have a water content of between 0.06% and 0.2% (Aissi et al., 2011; Codex Alimentarius, 2020). Except for butter derived from roasted kernels, the water content values of butter derived from other extraction methods are generally equal to or higher than those expected for grade II butter (Aissi et al., 2011; Ayegnon, Kayodé, et al., 2015). The latter could be used in soap factories or refined for better use (grades I or II) (Aissi et al., 2011). The low moisture content of food products is closely related to their long shelf life. Additionally, low moisture is desirable in fats and oils to preserve shelf life since low moisture prevents or reduces lipid oxidation, contamination, and microbial development. Nowadays, work is still being reported on the presence and variation of some nutrients, such as carbohydrates, crude protein, and the ash of *Pentadesma* butter. Also, no study investigated its microelement content. However, the current literature shows the fatty acid and triacylglycerol profiles of *Pentadesma* butter (Table 4). Thus, *Pentadesma* butter was characterized by different proportions of seven saturated or unsaturated fatty acids: palmitic, palmitoleic, stearic, oleic, linoleic, linolenic, and arachidic (Table 4). Several studies on the fatty acid composition have shown the predominance of stearic (C18:0) and oleic (C18:1ω9) acids, representing more than 96% of the total fatty acids. In contrast, the sterol fraction showed a predominance of ∆‐5 sterols, with stigmasterol being the main sterol. The triacylglycerol profile of *Pentadesma* butter was characterized by the presence of five triacylglycerols: palmityldioleylglycerol (POO), palmitylstearyloleylglycerol (POS), triolein (OOO), stearyldioleylglycerol (SOO), and distearyloleylglycerol (SOS) (Tchobo et al., 2007). SOS was the main triacylglycerol, with a concentration of over 50% (36.6%–62.9%), followed by SOO (33.6%–55.4%). Similarly, Honfo et al. (2014) observed the predominance of OOO (10.8%), SOO (35.2%), and SOS (40.4%) in shea butter. However, the values found were lower than those of *Pentadesma* butter. The butter's distinctive triglyceride composition, dominated by SOS and SOO, makes it easy to separate the high‐melting stearin fraction from the low‐melting olein fraction, and the resulting stearin fraction is then used in the cocoa butter equivalent blend (Alander, 2004). Despite the high content of stearic and oleic acids, Tchobo et al. (2007) reported a low content of triolein. Lately, Di Vincenzo et al. (2005) reported regional variation in the content of major triglycerides (SOO, OOO, and SOS) in shea butter.

**TABLE 4:** Overall chemical composition of *P. butyracea* butter.

Compounds	Content	References
Fatty acid (%)		
Palmitic acid	2–4	Ayegnon et al. (2015) Aissi et al. (2011) Tchobo et al. (2007) Dencausse et al. (1995) Kouadio et al. (1990) Adomako (1977)
Palmitoleic acid	0–0,2	
Stearic acid	37–48	
Oleic acid	47–59	
Linoleic acid	0.4–1.5	
Linolenic acid	0–0.5	
Arachidic acid	0–0.5	
Triacylglycerols (%)		
POO	0.1–0.6	Tchobo et al. (2007)
POS	0.8–2.3	
OOO	1.3–6.6	
SOO	33.6–55.4	
SOS	36.8–62.9	
Triterpenic alcohols (%)		
Butyrospermol	22.5	Dencausse et al. (1995)
β‐Amyrine	9.6	
Parkeol	3.6	
α‐Amyrine	39.7	
Lupeol	20.00	
Ψ‐taraxasterol	3.1	
Taraxasterol	1.5	
Sterols (%)		
Brassicasterol	3–6	Tchobo et al. (2007) Dencausse et al. (1995)
Campesterol	16–28	
Stigmasterol	44.7–69	
β‐Sitosterol	4–7	
∆^5^‐Avenasterol	0–1.6	
Spinasterol	0–11.4	
∆^7^‐Stigmasterol	0–11.0	
∆^7^‐Avénasterol	0–2.5	
Tocopherols (μg/g)		
α‐Tocopherol	29.2–97.4	Aissi et al. (2011) Tchobo et al. (2007)
β‐Tocopherol	8.3–107.8	
γ‐Tocopherol	3.1–18.4	
δ‐Tocopherol	9.3–31.1	

## 7 UNSAPONIFIABLE MATTERS

In the last decades, unsaponifiable plant oils and fats have been the subjects of intense research owing to their various bioactivities, including antioxidant, antimicrobial, anti‐inflammatory, and anti‐neuroinflammatory effects (Nahm et al., 2013; Siddique & Saleem, 2011; Toscano et al., 2019). The unsaponifiable matter content of *Pentadesma* butter (Table 5) was especially dominated by triterpene alcohols, followed by hydrocarbons, sterols, and other minor components such as tocopherol (Table 4). According to Dencausse et al. (1995), lupeol, ɑ‐amyrin, and butyrospermol are the main terpenic alcohols found in the unsaponifiable *Pentadesma* butter. These results agreed with the data reported by other authors (Akihisa et al., 2010; Honfo et al., 2014; Tom‐Dery et al., 2018), showing similar levels of the main terpenic alcohols in shea butter. The average sterol content of the *Pentadesma* butter was 1.773 μg/g oil (Tchobo et al., 2007). The phytosterol comprises ∆^5^ sterols (97%) (Tchobo et al., 2007). The two main phytosterols determined in the butter tree were stigmasterol (44.7%–69%) and campesterol (16%–28%). Brassicasterol and β‐sitosterol are sterols found in low concentrations. These phytochemical compounds are known for their significant biological activities. Indeed, previous studies have shown that these bioactive compounds could especially lower serum levels of low‐density lipoprotein (Honfo et al., 2014; Sharma et al., 2021). Contrary to *Pentadesma* butter, only two sterols, 7‐stigmasterol and stigmasterol, were found in shea butter (Honfo et al., 2014; Peers, 1977). Other minor bioactive compounds such as tocopherols were found in the unsaponifiable matter of *Pentadesma* butter. Generally, vegetable oils are an important source of tocopherols, so‐called vitamin E, which have free radical scavenging properties. According to Aissi et al. (2011), the tocopherol content of the *Pentadesma* butter varied with environmental factors and pretreatments undergone by the almonds. The total tocopherol levels in butter range from 95.3 to 194.7 μg/g (Aissi et al., 2011; Tchobo et al., 2007). They are made up of four isomers, especially α, β, γ, and δ‐tocopherols (Table 4). Among them, α‐ and β‐tocopherol were found in a high proportion (Aissi et al., 2011; Tchobo et al., 2007). In shea butter, tocopherols were extensively studied by several authors. Their findings revealed that the total tocopherol contents were more important and could reach 805 μg/g (Allal et al., 2013; Honfo et al., 2014; Maranz & Wiesman, 2004). According to Maranz and Wiesman (2004), the amount of α (and total) tocopherols increased in shea butter with an increasing mean regional temperature. Owing to their pivotal role in the nutrition and cosmetic industries, the presence of α‐tocopherol in shea and *Pentadesma* butter makes them an important product, especially in cosmetic applications, human nutrition, and health. Except for tocopherol (vitamin E), no referenced data report the vitamin contents of the butter of *Pentadesma*. However, the darker yellow color of *Pentadesma* butter might be due to the presence of carotenoids.

**TABLE 5:** Physicochemical characteristics of the *P. butyracea* butter and shea butter.

Parameter	*Pentadesma* butter	Shea butter
Melting point (°C)	32–39.5	32–39.5
Solidification point (°C)	22–29	20–30
Relative density (40°C)	–	0.9–1
Refractive index (40°C)	1.4–1.5	1.4–1.5
Saponification value (mg KOH/g)	185–194	132–207
Acid value (mg KOH/g)	0.3–8.3	0.4–21
Peroxide value (meq O_2_/kg)	0.83–1	0.5–29.5
Iodine value (mg I_2_/100 g)	47.3–48	21.7–89.5
Unsaponifiable content (%)	0.8–1.9	1.2–17.5
Free fatty acid (%)	0.49–2.37	1–10.7
Acidity (% oleic acid)	0.3–18	0.5–14.5
Impurity (%)	0.83–0.91	0.5–3.5
Color	Yellow	White to yellow

*Note*: Adapted from Adomako (1977), Kouadio et al. (1990), Dencausse et al. (1995), Tchobo et al. (2007), Aissi et al. (2011), Honfo et al. (2014), Ayegnon et al. (2015), Badoussi et al. (2022).

## 8 QUALITY CHARACTERISTICS OF *PENTADESMA* BUTTER

Physicochemical and sensorial characteristics such as moisture, free fatty acids (FFAs), insoluble impurities, iodine value, refractive index, melting point, peroxide values (PVs), and color (Table 5) were many parameters used to appreciate *Pentadesma* butter quality. However, according to Nsogning Dongmo et al. (2014), the most important quality criteria of plant butter are the FFA content and PV. The cosmetic industry uses first‐quality‐grade butter (FFA <1%, PV < 10 meq O_2_.kg^−1^). In contrast, second‐quality grade butter (FFA <3%, PV < 15 meq O_2_.kg^−1^) is appropriate for the food industry (Iddrisu et al., 2019). Peroxide is the first product of the oxidation of unsaturated fats and oils. According to Aissi et al. (2011), the PV of *Pentadesma* butter varies with pretreatment operations of kernels and butter, ranging from 0.83 to 1 meq O_2_ kg^−1^. Ayegnon et al. (2019) recently reported a PV of 2.7 meq O_2_ kg^−1^ for *Pentadesma* butter. These values were lower than those of shea butter (0.5–29.5 meq O_2_ kg^−1^) reported by several researchers (Dandjouma et al., 2009; Honfo et al., 2014) and also were lower than the maximum value of 10 meq O_2_ kg^−1^ required for cosmetic uses (Iddrisu et al., 2019; NBF 01‐005, 2006). The FFA content of *Pentadesma* butter is low and is in the range of 0.49%–2.37% (Table 5) (Jayathissa et al., 2023). The maximum threshold value of FFAs tolerated in cosmetics was 1%, against 3% for food uses (Aissi et al., 2011; Honfo et al., 2013; Iddrisu et al., 2019). The values of FFA found were much lower than those of shea butter (1%–10.7%) reported by most authors (Honfo et al., 2013, 2014; Nahm et al., 2013). According to Tom‐Dery et al. (2018), an FFA content of less than 7%, followed by low impurities (<1%), is currently tolerated in the markets. *Pentadesma* butter is in close agreement with these quality criteria since Aissi et al. (2011) found insoluble impurities ranging from 0.83% to 0.91%. The *Pentadesma* butter iodine values (47.3–48 mg of I_2_/100 g) reported by Dencausse et al. (1995) and Kouadio et al. (1990) were lower than those (64.3 and 89.5 mg of I_2_/100 g) obtained, respectively, by Adomako (1977) and Womeni et al. (2004) in shea butter. The unsaponifiables are liposoluble substances dissolved in fat. They are soluble in organic solvents after saponification. Unsaponifiable matter of *Pentadesma* butter determined by the Bolton and Williams' method varied between 1.5% and 1.8%, against 7.3% and 9.0% for shea butter (Adomako, 1977). However, concerning unsaponifiable, the maximum amounts are desirable in the cosmetics, personal care products, or pharmaceutical industries, while for the food industry, minimum amounts are generally relevant (Nahm et al., 2013). Moreover, Gbenga and Olaseni (2019) reported that the saponification value is inversely proportional to the mean molecular weight of the fatty acid in the glyceride present in the lipid. The saponification values of 185–194 mg KOH/g (Adomako, 1977; Aissi et al., 2011; Kouadio et al., 1990; Tchobo et al., 2013) were close to the reported values (132–207.5 mg KOH/g) by other researchers in shea butter (Honfo et al., 2014; Womeni et al., 2004). The refractive index is a key parameter that expresses the ratio between the speed of light in a vacuum and that of the substance under examination. In the case of oil, it is related to the degree of saturation and the ratio of double bonds (cis and trans). It can also give indications of oxidative alterations. In recent work, Ayegnon, Kayodé, et al. (2015) reported that this parameter was exploited in rapidly sorting fats and oils to assess their diversity and purity. The refractive indices at 40°C of *Pentadesma* (1.46) and shea (1.46) butter are quite similar (Aissi et al., 2011; Ayegnon, Kayodé, et al., 2015). According to Adomako (1977), *Pentadesma* and shea butter have identical melting points (39.0–39.5 and 38.0–39.5°C, respectively), and their solidification points (27.0–28.6 and 26.5–30°C, respectively) were not quite different. The relatively low melting point makes shea and *Pentadesma* butter solid or semi‐solid at room temperature. However, they melt when applied to the skin, making this butter a good body moisturizer with great spreading ability (Nahm et al., 2013). Moreover, the *Pentadesma* butter was light yellow in color, solid or semi‐solid at room temperature. It was found that there was a significant difference between the color of shea and *Pentadesma* butter, especially the yellow index (b*), which was significantly higher than that of the butter shea (Ayegnon, Kayodé, et al., 2015). According to these authors, the yellow color of *Pentadesma* butter was more attractive than shea butter. The butter of *P. butyracea* and shea are perfectly similar in many respects. However, the butter of *P. butyracea* possesses superior quality regarding organoleptic and chemical characteristics that give it a wide range of functional attributes (Ayegnon, Kayodé, et al., 2015; Badoussi et al., 2014).

## 9 HEALTH BENEFITS OF *PENTADESMA* BUTTER

No experimentally referenced data is available regarding the benefits of *Pentadesma* butter on human health. Nevertheless, some well‐documented surveys and ethnobotanical studies have usually reported the positive effect of *Pentadesma* butter in traditional practices. Thus, *Pentadesma* butter is extensively used in traditional medicine as a massage oil, in skin and hair care, and in the manufacture of soap for its softening, lubricating, and healing qualities (Dencausse et al., 1995). Like shea butter, *Pentadesma* butter might be a good moisturizer or emollient. Indeed, Ayegnon, Kayodé, et al. (2015) reported that a patented cosmetic product based on *Pentadesma* butter delayed skin aging. Skin‐rejuvenating properties of *Pentadesma* butter have also been reported. Phytosterols are an important part of the unsaponifiable fraction of vegetable oils. The hypocholesterolemia effects of phytosterol compounds are well recognized. Although there is no information on the use of unsaponifiable compounds from *Pentadesma* butter, Holanda Pinto et al. (2008) have shown that the triterpenes, α‐ and β‐amyrin extracted from *Protium heptaphyllum*, also present in *Pentadesma* butter, were effective in reducing the production of pro‐inflammatory cytokine TNF‐α related to acute periodontal inflammation. *Pentadesma* butter has also been employed in the treatment of several ailments. Maranz and Wiesman (2004) and Muotono and Maanikuu (2017) reported that the sun screening property of shea butter is related to shea butter's triterpenoids, especially cinnamate esters of triterpene alcohols that are known to have strong absorbance of ultraviolet radiation in the 250–300 nm range. These triterpenoid compounds were also present in *Pentadesma* butter and might have the same effect.

## 10 FUTURE PROSPECTIVES

*P. butyracea* is a multipurpose tree whose main product is butter extracted from its seeds. As in the case of shea butter, women are the main actors in the *Pentadesma* butter value chain. In many cases, *P. butyracea* butter is preferred for food and treatments (Ayegnon, Kayodé, et al., 2015). Due to this butter's nutritional quality and physicochemical and bioactive compounds, major food and cosmetic companies have filed over a dozen patents (Diel et al., 2022). In the past, *Pentadesma* seeds were exported to European countries. Its butter was extracted for various uses, including the partial or total substitution of cocoa butter (Diel et al., 2022) in confectionery and chocolate making due to its physicochemical properties similar to the latter. Unfortunately, this export has gradually fallen due to the decreasing availability of seeds over the years (Adomako, 1977). Thus, to develop the *Pentadesma* butter value chain for better access to national, regional, and international markets, much effort needs to be made by public authorities toward local communities in the countries concerned. First, using the tree's wood as a building or raw material for charcoal production seriously jeopardizes the species. This form of exploitation of *P. butyracea* must be banned. As local communities are the main beneficiaries of a possible *Pentadesma* butter boom, raising awareness would help quickly remove this obstacle and improve their productivity. If necessary, enforcement of deforestation laws could deter violators. Given that the butter comes from the seed, the quality of the constituent fatty acids as well as the technical characteristics of the butter could depend, to a large extent, on the species and cultivars of this plant. Generally, there needs to be more information on the genetic diversity of the species and the morphological variability of *P. butyracea* seeds, about their fat content, and technological abilities. Data combining physicochemical, morphological, and molecular features would be paramount in a dynamic of identification, selection, and promotion through the domestication of better quality genotypes. Thus, studies on domestication, genetic diversity evaluation, and species productivity will be undertaken. At the same time, recognizing the tree's potential value in reforestation programs will sustainably promote this resource's development.

Moreover, more data are needed on transforming *P. butyracea* seeds into butter. The traditional processing is similar to that of shea butter production. The only difference is that the shea nuts are dehulled, as *P. butyracea* kernels do not have hulls (Aissi et al., 2011). This butter, like many native kinds of butter of African origin, is often obtained through traditional pre‐treatment and extraction processes. Traditional pre‐treatments such as boiling, roasting (Figure 3a), frying, or smoking fresh seeds have been reported to ensure their preservation. However, they significantly influence their physicochemical characteristics, extraction rate, and butter quality. The extraction from boiled and dried seeds is more difficult but gives the highest yield (Aissi et al., 2011; Badoussi et al., 2014). Butter from traditionally roasted seeds has the lowest acidity, water, and volatile matter contents. It has been observed that α‐linolenic acid and β‐ and δ‐tocopherol are completely lost after post‐harvest pretreatments (Aissi et al., 2011). The processing equipment design to move from the traditional exclusively manual scale to the mechanical scale with an optimized process would be a major innovation leading to standardized premium butter. A recent investigation has shown that the processing technology and packaging type significantly affect butter's physicochemical and microbiological properties (Badoussi et al., 2022). For this reason, innovation will also consider the butter package material.

Regarding folk medicinal use of the plant and the health claims reported by ethnobotanical investigations, further ethnopharmacological studies will have to be undertaken to identify new bioactive compounds for the development of drugs. Therefore, it is urgent to domesticate the butter tree and incorporate it into agroforestry systems in its natural areas. Domestication will also significantly reduce pressure on natural forest populations. This will contribute to improving food security and reducing poverty by increasing the livelihoods of rural communities.

## 11 CONCLUSION

*Pentadesma butyracea* is one of the main multipurpose trees native to tropical Africa. This species is recognized for its economic, nutritional, socio‐cultural, cosmetic, and pharmaceutical benefits. It has significant economic potential, and its sustainable management will contribute to food security by empowering women and increasing their income. However, appropriate measures must be taken into account in order to avoid these innovations and improve market access for butter tree products, leading to overexploitation of the species. Sustainability plans are to be established to avoid the anarchical cutting of the *P. butyracea* tree. Expansion policies must be adopted, and new performant varieties must be introduced.

## INSTITUTIONAL REVIEW BOARD STATEMENT

Not applicable.

## INFORMED CONSENT

Not applicable.

## AUTHOR CONTRIBUTIONS

**Ifagbémi Bienvenue Chabi:** Data curation (equal); formal analysis (equal); funding acquisition (equal); investigation (equal); methodology (equal); software (equal); supervision (equal); visualization (equal); writing – original draft (equal); writing – review and editing (equal). **Midimahu Vahid Aïssi:** Data curation (equal); funding acquisition (equal); investigation (equal); resources (equal); supervision (equal); validation (equal); visualization (equal). **Oscar Zannou:** Funding acquisition (equal); investigation (equal); methodology (equal); resources (equal); software (equal). **Yénoukounmè E. Kpoclou:** Formal analysis (equal); funding acquisition (equal); investigation (equal); methodology (equal); supervision (equal); validation (equal); visualization (equal); writing – original draft (equal). **Bernolde Paul Ayegnon:** Funding acquisition (equal); investigation (equal); methodology (equal); project administration (equal); supervision (equal); validation (equal). **Marius Eric Badoussi:** Data curation (equal); formal analysis (equal); funding acquisition (equal); investigation (equal); software (equal); visualization (equal). **Vénérande Y. Ballogou:** Data curation (equal); formal analysis (equal); investigation (equal); resources (equal); validation (equal); visualization (equal). **Gulden Goksen:** Formal analysis (equal); investigation (equal); project administration (equal); software (equal); supervision (equal); validation (equal); writing – original draft (equal); writing – review and editing (equal). **Amin Mousavi Khaneghah:** Data curation (equal); formal analysis (equal); funding acquisition (equal); supervision (equal); writing – original draft (equal); writing – review and editing (equal). **Adéchola P. Polycarpe Kayodé:** Conceptualization (equal); funding acquisition (equal); investigation (equal); methodology (equal); resources (equal); supervision (equal); validation (equal); writing – original draft (equal).

## CONFLICT OF INTEREST STATEMENT

The authors declare no conflict of interest.

## Data Availability

All data generated or analyzed during this study are included in this published article.
